# Grand Challenge: On the Way to Scarless Visceral Surgery

**DOI:** 10.3389/fsurg.2014.00011

**Published:** 2014-04-17

**Authors:** Ferdinand Köckerling

**Affiliations:** ^1^Department of General Surgery and Center of Minimally Invasive Surgery, Vivantes Hospital Berlin, Academic Teaching Hospital of Charité Medical School, Berlin, Germany

**Keywords:** minimally invasive surgery, natural orifice transluminal endoscopic surgery, robotic surgery, mini-laparoscopy, mono-port

The ability to conduct operative procedures in the abdominal cavity with minimal or no scarring has been a dream long cherished by mankind. Therefore, it is not surprising that minimally invasive surgery spread across the world with remarkable speed after its introduction some 25 years ago. The patient has smaller incisions, faster recovery time, spends less time in hospital, and the costs are also reduced. Surgeons rapidly learned the techniques, something demanded by patients (1).

Using the standard laparoscopic technique, the procedure is performed in the abdomen with three to four trocars measuring 5–12 mm in diameter. Meta-analyses have in the meantime demonstrated that in the majority of visceral surgery procedures, the standard laparoscopic technique has advantages for the patient compared with the open technique.

Standard laparoscopic cholecystectomy has advantages over the open operation regarding duration of hospital stay and convalescence (2).

Standard laparoscopic appendectomy provides considerable benefits over appendectomy, including a shorter length of hospital stay, less postoperative pain, earlier postoperative recovery, and a lower complication rate (3, 4).

Standard laparoscopic anti-reflux surgery assures faster convalescence and return to productive activity compared with open surgery, with a reduced risk of complications and similar treatment outcome (5, 6). Standard laparoscopic bariatric surgery is a safer method of treatment than open surgery (7). Endoscopic repair of inguinal hernias has significant advantages in terms of pain-associated parameters in comparison to open techniques (8, 9). In a meta-analysis, elective standard laparoscopic sigmoid colectomy for diverticular disease showed lower overall morbidity, earlier return to bowel function, and shorter hospital stays than open surgery (10).

Accordingly, the standard laparoscopic technique currently represents the gold standard in visceral surgery for several benign diseases.

Although the risks are higher when using minimally invasive surgery for malignancies of intra-abdominal organs, this technique is also being used in tumor surgery (11). Obviously, the laparoscopic approach can only be implemented in cases of early, small sized, cancers confined to the target organ (11). In the hands of experienced surgeons and with appropriate patient selection, use of the standard laparoscopic technique to treat colorectal cancer and gastric cancer can achieve long-term oncological results comparable with those of the open technique, with lower postoperative complication rates (12–14).

Despite the improvements in outcomes with laparoscopy, the technique has limitations (15), such as two-dimensional imaging, restricted range of motion of the instruments, and poor ergonomic positioning of the surgeon (16). The robotic surgery system was introduced as a solution to minimize the shortcomings of laparoscopy (17). Robotic systems have 3D imaging, tremor filter, and articulated instruments (18). With this advanced equipment, robotic surgery is superior to conventional laparoscopic surgery (19). Even for demanding visceral surgery procedures, the perioperative complication rate for robotic surgery is not higher than for open or standard laparoscopic surgical procedures. In cancer cases, the oncological accuracy of robotic resection for gastric, pancreatic, and rectal resection is seen to be adequate (16, 20, 21). To evaluate the future role of the robotic technique for visceral surgery, high-quality prospective randomized trials are urgently needed.

Whereas robotic surgery is aimed at improving and optimizing the technical feasibility of standard laparoscopic operations, mini-laparoscopy, single-port technique, and natural orifice transluminal endoscopic surgery (NOTES) endeavor to further reduce access trauma to the abdominal wall, or shift this to the natural orifices in hollow organs (stomach, vagina, bladder, intestines).

Using the mini-laparoscopic technique, 5–12 mm trocars are partially replaced with 2 mm trocars, thus reducing the overall length of the incisions. A meta-analysis and systematic review of mini-laparoscopic versus standard laparoscopic cholecystectomy showed improved cosmesis and quicker return to activity (22).

For the mono-port or single-incision technique, three to four trocars measuring 5–12 mm in diameter are replaced with a larger trocar with a diameter of 2–3 cm. The latter is generally placed in the region of the “natural scar,” i.e., the navel. This trocar has three to four working channels for the instruments and optics. Meta-analyses of prospective randomized studies of single-incision versus standard laparoscopic cholecystectomy showed a higher conversion rate, longer operating time, and greater blood loss for the single-port technique. However, the cosmetic results were better too (23, 24).

To date, the clinical access routes used in NOTES have been the stomach, vagina, and rectum. Up to August 2012, reports on 1,200 NOTES procedures have been published in the literature (25). Transvaginal cholecystectomy is the commonest NOTES procedure reported, and its clinical feasibility and safety were established through prospective case series and randomized trials (25). The transvaginal access route was also used for extraction of numerous organs, including appendix, kidney, bladder, and colon (26). NOTES represents the latest promising technology that allows new access into cavities of the human body, especially the abdominal cavity. It has generated excitement among physicians for a potentially scar-free surgery (26). Most of the approaches seem to be feasible but to achieve better results and wider application to human beings, new endoscopic instruments need to be designed to facilitate each approach.

New-concept instruments need to be developed for NOTES, among which robotics provide a promising way forward. The feasibility of using operative and imaging micro-robots for task assistance has been validated in a pilot study. As more advanced robotic systems are being proposed and developed, so it can be expected that NOTES procedures will become more mature and more widely accepted in the future (27).

Accordingly, further technical developments will determine what role NOTES, and hence the scarless approach, will play in visceral surgery. Of paramount importance will be how well flexible endoscopy can be adapted to meet the demands of NOTES and its integration into robotic systems. However, the rapid pace of technical advancements witnessed in minimally invasive surgery over the past 25 years has demonstrated that such technical innovations will become reality, thus ushering in the era of scarless visceral surgery.

## Conflict of Interest Statement

The author declares that the research was conducted in the absence of any commercial or financial relationships that could be construed as a potential conflict of interest.

## References

[1] Schimpff SC The Future of Medicine: Megatrends in Healthcare that will Improve your Quality of Life. Nashville, TN: Thomas Nelson Inc. (2007).

[2] Keus F Gooszen HG van Laarhoven CL. Systematic review: open, small-incision or laparoscopic cholecystectomy for symptomatic cholecystolithiasis. Aliment Pharmacol Ther (2009) 29:359–78. 10.1111/j.1365-2036.2008.03894 19035965

[3] Li X Zhang J Sang L Zhang W Chu Z Li X Laparoscopic versus conventional appendectomy – a meta-analysis of randomized controlled trials. BMC Gastroenterol (2010) 10:129. 10.1186/1471-230X-10-129 21047410 PMC2988072

[4] Wei B Qi CL Chen TF Zheng ZH Huang JL Hu BG Laparoscopic versus open appendectomy for acute appendicitis: a meta-analysis. Surg Endosc (2011) 25:1199–208. 10.1007/s00464-010-1344-z 20848140

[5] Peters MJ Mukhtar A Yumus RM Khan S Pappalardo J Memon B Meta-analysis of randomized clinical trials comparing open and laparoscopic anti-reflux surgery. Am J Gastroenterol (2009) 104:1548–61. 10.1038/ajg.2009.176 19491872

[6] Siddiqui MRS Abdulaal Y Nisar A Ali H Hasan F A meta-analysis of outcomes after open and laparoscopic Nissen’s fundoplication in the treatment for gastro-oesophageal reflux disease. Eur Surg (2012) 44:138–49. 10.1007/s00383-010-2698-y 20734053

[7] Reoch J Mottilo S Shimony A Filion KB Christou NV Joseph L Safety of laparoscopic vs open bariatric surgery. Arch Surg (2011) 146:1314–22. 10.1001/archsurg.2011.270 22106325

[8] Bittner R Sauerland S Schmedt CG. Comparison of endoscopic techniques vs Shouldice and other open nonmesh techniques for inguinal hernia repair: a meta-analysis of randomized controlled trials. Surg Endosc (2005) 19:605–15. 10.1007/s00464-004-9049-9 15789255

[9] Schmedt CG Sauerland S Bittner R. Comparison of endoscopic procedures vs Lichtenstein and other open mesh techniques for inguinal hernia repair: a meta-analysis of randomized controlled trials. Surg Endosc (2005) 19:188–99. 10.1007/s00464-004-9126-0 15578250

[10] Siddiqui MR Sajid MS Khatri K Cheek E Baig MK. Elective open versus laparoscopic sigmoid colectomy for diverticular disease: a meta-analysis with the Sigma trial. World J Surg (2010) 34:2883–901. 10.1007/s00268-010-0762-3 20714895

[11] Veronesi U Stafyla V Grand challenges in surgical oncology. Front Oncol (2012) 2:127 10.3389/fonc.2012.00127 23061044 PMC3462322

[12] Chen K Xu XW Zhang RC Pan Y Wu D Mou YP. Systematic review and meta-analysis of laparoscopy-assisted and open total gastrectomy for gastric cancer. World J Gastroenterol (2013) 19:5365–76. 10.3748/wjg.v19.i32.5365 23983442 PMC3752573

[13] Ma Y Yang Z Qin H Wang Y. A meta-analysis of laparoscopy compared with open colorectal resection for colorectal cancer. Med Oncol (2011) 28:925–33. 10.1007/s12032-010-9549-5 20458560

[14] Trastulli S Cirocchi R Listorti C Cavaliere D Avenia N Gullà N Laparoscopic vs open resection for rectal cancer: a meta-analysis of randomized clinical trials. Colorectal Dis (2012) 14:e277–96. 10.111/j.1463-1318.2012.02985.x 22330061

[15] Harrel AG Heniford BT. Minimally invasive abdominal surgery: lux et veritas past, present, and future. Am J Surg (2005) 190:239–43. 10.1016/j.amjsurg.2005.05.019 16023438

[16] Marano A Choi YY Hyung WJ Kim YM Kim J Noh SH. Robotic versus laparoscopic versus open gastrectomy: a meta-analysis. J Gastric Cancer (2013) 13:136–48. 10.5230/jgc.2013.13.3.136 24156033 PMC3804672

[17] Aggarwal R Hance J Darzi A Robotics and surgery: a long-term relationship? Int J Surg (2004) 2:106–9 10.1016/S1743-9191(06)60055-1 17462231

[18] Sodergren MH Darzi A Robotic cancer surgery. Br J Surg (2013) 100:3–4 10.1002/bjs.8972 23132653

[19] Liao G Chen J Ren C Li R Du S Xie G Robotic versus open gastrectomy for gastric cancer: a meta-analysis. PLoS One (2013) 8:e81946. 10.1371/journal.pone.0081946 24312610 PMC3849388

[20] Chen Y Yan J Yuan Z Yu S Wang Z Zheng Q. A meta-analysis of robotic-assisted pancreatectomy versus laparoscopic and open pancreatectomy. Saudi Med J (2013) 34:1229–36. 24343462

[21] Trastulli S Farinella E Cirocchi R Cavaliere D Avenia N Sciannameo F Robotic resection compared with laparoscopic rectal resection for cancer: systematic review and meta-analysis of short-term outcome. Colorectal Dis (2012) 14:e134–56. 10.1111/j.1463-1318.2011.02907.x 22151033

[22] Thakur V Schlachta CM Jayaraman S. Mini-laparoscopic versus conventional laparoscopic cholecystectomy: a systematic review and meta-analysis. Ann Surg (2011) 253:244–58. 10.1097/SLA.0b013e318207bf52 21183848

[23] Trastulli S Cirocchi R Desiderio J Guarino S Santoro A Parisi A Systematic review and meta-analysis of randomized clinical trials comparing single-incision versus conventional laparoscopic cholecystectomy. Br J Surg (2013) 100:191–208. 10.1002/bjs.8937 23161281

[24] Sajid MS Ladwa N Kalra L Hutson KK Singh KK Sayegh M Single-incision laparoscopic cholecystectomy versus conventional laparoscopic cholecystectomy: a meta-analysis and systematic review of randomized controlled trials. World J Surg (2012) 36:2644–53 10.1007/s00268.012-1719-5 22855214

[25] Liu L Chiu PWY Reddy N Ho KY Kitano S Seo DW Natural orifice transluminal endoscopic surgery (NOTES) for clinical management of intra-abdominal diseases. Dig Endosc (2013) 25:565–77. 10.1111/den.12154 23967798

[26] Moris DN Bramis KJ Mantonakis EI Papalampros EL Petrou A Papalampros AE. Surgery via natural orifices in human beings: yesterday, today, tomorrow. Am J Surg (2012) 204:93–102. 10.1015/j.amjsurg.2011.05.019 22206853

[27] Wang X Meng MQH Robotics for natural orifice transluminal endoscopic surgery: a review. J Robot (2012) 2012:512616 10.1155/2012/512616

